# Ten simple rules for organizing a special session at a scientific conference

**DOI:** 10.1371/journal.pcbi.1010395

**Published:** 2022-08-25

**Authors:** Davide Chicco, Philip E. Bourne

**Affiliations:** 1 Institute of Health Policy Management and Evaluation, University of Toronto, Toronto, Ontario, Canada; 2 School of Data Science, University of Virginia, Charlottesville, Virginia, United States of America; Carnegie Mellon University, UNITED STATES

## Abstract

Special sessions are important parts of scientific meetings and conferences: They gather together researchers and students interested in a specific topic and can strongly contribute to the success of the conference itself. Moreover, they can be the first step for trainees and students to the organization of a scientific event. Organizing a special session, however, can be uneasy for beginners and students. Here, we provide ten simple rules to follow to organize a special session at a scientific conference.

## Introduction

Scientific conferences are events where researchers, students, and scholars gather to present and discuss new scientific developments and results regarding a specific field. Conferences are sometimes called *symposiums* or *scientific meetings*, as well. A typical bioinformatics conference includes special sessions (sometimes called *special tracks*) that are parts of the conference dedicated to a specific subfield or to a particular topic. In this manuscript, we consider special sessions where (short) scientific papers are submitted by the authors, reviewed, and selected for inclusion in the special session. The authors of the accepted articles usually have then a slot to present their work as a plenary talk (if the article’s quality is outstanding), as a short talk (if the article’s quality is good), or as a scientific poster (if the article’s quality is sufficient), based on the assessment of the reviewers or of the special session’s organizers.

Other scientific meetings call *special sessions* brainstorming discussions on new and emerging fields where participants are engaged in an active dialog and ideas exchange, without peer-reviewed papers. In this manuscript, we refer to the former definition. Some conferences use the term *workshop* as a synonym of *special session*. In our experience, workshops are satellite events of conferences, related to the scientific meeting but actually happening outside its rooms and schedule. Special sessions, instead, happen within scientific conferences. Regarding duration, we believe a successful special session can occupy half a day of a conference schedule, even if this allocated time can be bigger (in case of many accepted papers) or smaller (in case of few accepted articles).

An accepted special session must follow the format of their main conference, of course: If the main conference has a unique main track, the special session will occupy a part of the program of the main track. If the conference has several tracks in parallel, instead, the special session will happen during one of them.

In the past, the PLOS Computational Biology Ten Simple Rules collection already provided guidelines for organizing scientific meetings [1], virtual conferences [2,3], unconferences [4], for curating and facilitating small workshops [5], and for chairing special sessions at conferences [6], but did not give suggestions on how to organize special sessions. We fill this gap here by providing this short list of simple rules learnt by organizing special sessions during the International Meeting on Computational Intelligence Methods for Bioinformatics and Biostatistics (CIBB) [7–9] and other occasions.

### Rule 1: Pick a specific, cutting-edge topic

The topic of the special session is a pivotal pillar of the success of the special session itself. It should not be too broad (for example, “Convolutional neural networks in epigenetics”), but rather specific to a subfield of the scientific domain of the main conference. Moreover, the topic should be new and cutting-edge: something that is getting momentum in the scientific literature. Ideally, your special session talks should contribute to answering important scientific questions in the field. The title of the special session, additionally, should be clear and concise, without jargon or too many technical words. Moreover, in bioinformatics and medical informatics, focusing the special session on a particular disease, such as heart attack [10] or amyotrophic lateral sclerosis (ALS) [11], could be a good idea.

Choosing a data type for the studies of the special session could be another efficient way to define the special session topic: You and your co-organizers might decide to limit the special session to data of electronic health records [12,13], radiomics images [14], electrocardiogram (ECG) signals [15], microarray gene expression [16], RNA-seq gene expression [17,18], biomolecular annotations [19], or epigenomics [20], for example. Alternatively, the special session can be focused on a specific method, such as functional enrichment analysis [21], bioinformatics data clustering [22], or multiomics data integration [23,24].

Another aspect to consider are the possible competing special sessions: Check the websites of the previous editions of the conference and see if there are recurring special sessions organized for several editions of the conference in the past. If there, are they on the same scientific topics of the special session you plan to propose? If yes, then it would be better to change subjects, or to ask them to enter their organizing committee. If no, feel free to move on with the scientific topics you picked. Do the same check regarding special sessions of other scientific meetings: If there might be overlap with the special session of another conference, consider changing subjects.

### Rule 2: Find some reliable co-organizers among your collaborators

A key asset for the organization of a special session (and of anything in life) is the collaborators that will help you. You need to choose reliable people. Not the smartest, fastest, popular people. For reliable, we mean individuals who will respect their commitments on time: If they agree to do X before the time Y of the day Z, you can rest assured they will do it on time. A mix of junior collaborators and senior collaborators can be the best solution. Moreover, the principles of diversity, equity, and inclusion should be considered in the creation of the organizing committee, too. The special session organizers should look for people with different conditions (in body ability, career stage, gender, gender identity, gender expression, geographic origin, language, neurodiversity, political views, race, religion, sexual orientation, and socioeconomic background [25]) to be included in the organizing committee. Particular attention should be paid for gender balance [26].

The main task to assign to collaborators is the spread of the call for papers (Rules #6 and #7): The number of articles submitted is the single most important aspect for having a successful special session, so all the co-organizers should put their effort into this activity. This rule goes arm in arm with the sixth rule of the ten simple rules for organizing a scientific meeting by Corpas and colleagues (“Carefully select your key helpers: the organizing committees”) [1]: We suggest you follow it, too. Additionally, having regular meetings with the rest of the team (for example, every two weeks) can be a very useful practice.

### Rule 3: Find a sponsor for financial support, if necessary

Having enough funds to organize an event is always an important aspect. A special session might not need extra funding and therefore might not require extra financial support. In some cases, however, additional expenses not covered by the main conference budget might be there, for example, if the special session organizers want to invite a keynote speaker (Rule #4). In this case, we suggest calculating how much money you need for these extra costs, and to contact companies or scientific societies asking them to sponsor your special session. Of course, companies should be in the same technological area as the theme of your special session and should be approached only if they are not already sponsoring that specific conference.

In return, one can offer the possibility to insert their banner and web link on the special session website (Rule #5), and/or the chance to have a booth at the conference during your special session’s day. In case of involvement of companies, remember to take into account the possible conflicts of interests, which must all be declared in advance. One might also ask for financial support from a scientific society, such as the national bioinformatics association of the country where the conference is organized, for example. Sponsor’s funds can also be used to secure travel fellowships for undergraduates, graduate students, and postdocs to help them attend the conference. This would encourage the participation of people coming from low-income countries.

### Rule 4: Invite a keynote speaker on the subject

Having a well-known researcher or professor giving a keynote talk during a special session can make the real difference between a successful and an unsuccessful special session. As Peter Jansson explained on Academia Stack Exchange, having a keynote speaker famous in the special session field would gain interest from people who know her/him and give credibility to the special session [27]. The keynote speaker should be invited to talk about a scientific study related to the special session themes, of course. Young principal investigators and team leaders might be more available to accept an invitation than senior professors.

If you have enough funds (Rule #3), you and your co-organizers might even consider inviting two keynote speakers instead of one. In this case, it would be better to have one keynote talk at the beginning of the special session and the other one at the end. Regarding the order, we suggest to put the more general, wide talk on the scientific topic at the start, so it can also serve as an introduction to the subject. And we recommend to put the more innovative, cutting-edge talk at the end, so that attendees leave the special session with a glimpse on the future directions of the scientific field in their mind. Again, it is important to reaffirm that the principles of diversity, equity, and inclusion should be kept in mind when selecting potential keynote speakers [25,26,28]. Some practical steps to this end include looking for speakers far beyond your personal network and contacting relevant professional society chapters of underrepresented minorities [29].

Underrepresented individuals should be invited first. It is important to identify excellent investigators who may have not yet had the exposure of keynotes in the past.

### Rule 5: Release a website on the special session

The scientific conference of the special session surely will have a website with all the scientific, technical, and logistic information: Even if useful, its large amount of information might obscure special sessions, including yours. A good idea here is to implement and release a website about your special session that you can include when you share the call for papers among collaborators and colleagues.

The website should be simple, with clear information about how to submit papers and how to contact the special session organizers, and built following accessibility guidelines [30]. This special session website should be linked from the main conference website and contains a web link to it, of course.

After the conference, you can use the website to spread information about the scientific contents of your special session: You can insert the titles and abstracts of the presented articles and slides of the talks, for example. This way, you will make the impact of the special session last longer.

### Rule 6: Spread the call for papers on community websites, newsletters, mailing lists, and social media

Another key point of a successful special session is the spread of the call for papers. The more people get to know about your special session, the more authors can decide to submit a paper, and the more papers your special session will receive. We suggest you to spread your call for papers via email through mailing lists and newsletters of groups of researchers interested in the special session themes, at least two months before the papers’ submission deadlines. University newsletters and websites, topic mailing lists, channels of scientific societies and informal groups, your personal website: Anything can help.

Beginners and students can ask advice to senior professors and researchers on which mailing lists and newsletters are more popular and established in the special session’s scientific field. Again, particular importance should be given to reaching underrepresented minorities and groups [25,26].

Of course, the call for papers should be announced on social media as well: Twitter, in particular, has become very popular in many scientific communities in the last ten years. A Twitter account related to the special session, sending regular messages about the call for papers, can be a useful asset. Asking for help to Twitter users having more popular, visible Twitter accounts can be effective, too.

### Rule 7: Contact potential authors directly via email and invite them to submit an article

Another good way to spread the call for papers is contacting potential authors individually via email. If you know about people working on the special session topic, who might be interested in submitting a paper, you can consider contacting each of them directly informing them about the open call for papers. Moreover, this is a great occasion to invite colleagues and coworkers of your department and institution to submit an article. Oftentimes we are more informed about what happens in online scientific communities than about what happens in our university departments: A special session can be an opportunity to involve colleagues and coworkers towards a common event.

Additionally, directly contacting colleagues and coworkers can give you a bit of visibility that might help you in your career in the future. Again, the principles of diversity, equity, and inclusion should be considered when contacting potential paper authors and participants [25,26]. Once this phase on the spread of call for papers has finished, discuss with your co-organizers how many articles you expect to receive and how many attendees you expect to see. Having a foreseen number is the key: Knowing if you and your co-organizers should expect 5 or 100 articles, 10 or 200 attendees can make a big difference in your expectations.

This information will allow you to forecast important details such as how many reviewers you will need to handle the submitted papers’ review phase, how much time your special session will occupy in the conference schedule, how much time can be allocated for each paper talk, how large the conference room needs to be for your special session, and approximately how many extended papers will be submitted to the special session special issue on a journal (Rule #9).

Predicting the approximate number of submitted articles is tricky; a hint can come from the previous conference editions. The organizers of the previous conference editions can provide this precious information.

### Rule 8: During the special session, follow the “Ten simple rules for chairing a scientific session” by Alex Bateman and Philip E. Bourne

All our previous rules refer to what to do before the conference. On the special session day, you and your co-organizers will have to chair the special session, making sure everything works well. To this end, we simply suggest you follow the ten guidelines for chairing a scientific session by Alex Bateman and Philip E. Bourne [6]. As the authors clearly explain, timing is the key: Do not let things overrun, write down the actual start times of the speakers, do have a watch, communicate how much time is left to the speaker, and keep control of the question and answer sessions [6].

For in-person conferences, we really like the Rule #8 about getting to the venue early in advance to test and check all the technological devices (laptop, microphone, projector, etc.). We believe this rule should be kept in mind not only for special sessions but also for any lecture or class.

If the conference is virtual, tests on the online conference platform should be done in advance, too. Additionally, we recommend organizers to ask speakers to give their presentations and talks following accessibility guidelines [30].

### Rule 9: Arrange a journal special issue for extended versions of the special session accepted papers

A good way to make the impact of your special session last is to organize a special issue of a scientific journal accepting only submitted articles that are extended versions of the papers presented at the special session. This way, authors can take advantage of both the reviewers’ comments obtained during the article peer review and of the participants’ feedback received during the special session and improve their articles making them ready for a journal publication.

A special issue is a collection of articles related to a specific topic or to a specific event. These collections can have different names: *Special issue* is the name used by IEEE journals and Oxford University Press (OUP) journals, while the BioMed Central journals call them *supplements* and the Frontiers Media journals call them *research topics*. The organizers of the special session usually serve as guest editors for these collections. Guest editors usually handle the first review phase of these manuscripts (inviting reviewers, collecting reviewers’ reports, making recommendations and decisions, etc.), eventually making a final recommendation for the acceptance or the rejection of each article. After this first review phase, an associate editor-in-chief of the journal receives and studies all the review materials of the first phase and decides if recommending the article for acceptance or rejection, or if starting a new review phase with new reviewers. The publication of the special issue in an international scientific journal gives credibility to the special session, especially for the future editions.

This special issue should be published after the conference, but its arrangement should start months before it. The organizers have to contact and negotiate an agreement with the aimed journal at least six months before the special session day. The first step is simply selecting a respectable journal on the special session subjects and then contacting the editorial board via email, explaining your intentions. Chances of proposal acceptance are higher for journals that already published conference special issues in the past, of course. Once the agreement with the journal is finalized, the special issue should be advertised on the special session website and in the call for papers: This way, more authors will be attracted and more articles will be submitted. The quality of the manuscripts will also be higher.

Several bioinformatics conferences publish post-conference supplements in international journals: for example, the ECCB and ISMB conferences in the Bioinformatics (Oxford) journal [31]; NETTAB and BITS in BMC Bioinformatics [32,33]; CIBB in BMC Medical Informatics and Decision Making [34] and BMC Bioinformatics [35]; ACM BCB [36], GENSIPS [37], BIBM [38], BIOKDD [39], DMBIH [40], and others in IEEE/ACM Transactions on Computational Biology and Bioinformatics; VIZBI in Frontiers in Bioinformatics [25]; iABC in Journal of Integrative Bioinformatics [41]; and AIME in Artificial Intelligence in Medicine [42]. Some conference organizers or participants also publish conference reports to highlight and recap the main insights of the organized scientific meetings [43]: It is a possibility that we suggest to consider, too.

### Rule 10: If everything went well, organize the special session again during the next conference edition

After months of work, email exchange, online meetings, in-person meetings, and many other unexpected things, the day of your special session arrives: Everything goes well, and, fortunately, you can consider this special session successful. For the next conference edition, if the stress and the fatigue of your special session organization were bearable, you might consider to treasure your experience and organize the special session again.

You surely will have learnt which aspects to improve and what changes to make for the next edition, and probably you will have new collaborators available to help you. For the following edition, you can also consider to leave the leadership role and to pass it on to other collaborators, perhaps junior members.

You would supervise their work, of course, but this way you would let them gain some precious experience.

## Conclusions

Special sessions are the core of scientific conferences: They bring together researchers, students, and scholars investigating a particular subject, facilitating debates, ideas exchanges, and collaborations on that topic. Most scientific conferences used to be in-person, but, recently, virtual conferences and hybrid conferences (partially presential and partially virtual) emerged worldwide, due to COVID-19 pandemic.

We originally designed these ten simple rules for in-person meetings, but we believe they are valid for virtual and hybrid conferences, too.

Regarding timing, the decision to organize a special session should be made at least one year in advance with respect to the conference dates, to allow enough time for preparation. Some conferences, such as ISMB ECCB, for example, have a specific deadline for the special session proposal submissions; these deadlines must be met, of course. Another important aspect to keep in mind is documentation: You might record details and information about all your efforts and tasks of the special session organization in a diary, from the first organizing day to the special session day. This diary will then be useful for organization of the next special session edition and can be shared with new collaborators.

All in all, organizing a special session can be a tiresome activity for researchers, especially for people with a busy schedule. We hope these ten simple rules can help anyone around the world organize great, interesting special sessions at scientific meetings. To the ones doing it for the very first time: It is likely that something will go wrong, but that is completely okay, as long as you are willing to learn from your mistakes and misadventures. As the Cult of Done Manifesto says: “People without dirty hands are wrong. Doing something makes you right” [44]. So, whatever happens, keep in mind the quote by Sheryl Sandberg: “Done is better than perfect.”
